# Prevalence and Prognostic Significance of Extramural Venous Invasion in Patients with Locally Advanced Esophageal Cancer

**DOI:** 10.1245/s10434-018-6448-z

**Published:** 2018-04-02

**Authors:** Zohra Faiz, Lotte J. W. Huijgen, H. J. Alqethami, J. G. M. Burgerhof, Gursah Kats-Ugurlu, John T. M. Plukker

**Affiliations:** 1 Department of Surgery, University of Groningen, University Medical Center Groningen, Groningen, The Netherlands; 2 Department of Pathology, University of Groningen, University Medical Center Groningen, Groningen, The Netherlands; 3 Department of Epidemiology, University of Groningen, University Medical Center Groningen, Groningen, The Netherlands

## Abstract

**Background:**

Extramural venous invasion (EMVI) is a known adverse prognostic factor in patients with colorectal carcinoma. The prevalence and significance of EMVI in esophageal cancer (EC) patients is still unclear.

**Methods:**

From a prospectively maintained database, we retrospectively reviewed the resection specimens of patients with pathologic locally advanced (pT3/T4/N0-3) EC who were treated with curative intent between 2000 and 2015. Patients with previous malignancies and gastroesophageal junction (type II/III) tumors were excluded. Included were 81 patients who underwent surgery alone and 37 patients who underwent neoadjuvant chemoradiotherapy (nCRT). EMVI was assessed on hematoxylin and eosin slides and confirmed or excluded by additional Elastica van Gieson staining. Survival was analyzed using a multivariable Cox regression.

**Results:**

EMVI was present in 23.5% (*n* = 19) of patients in the surgery-alone group and 21.6% (*n* = 8) of patients in the nCRT group. The prevalence of EMVI after surgery alone was significantly high in squamous cell carcinomas and among tumors located in the mid-esophagus, as well as those with lymphovascular invasion (*p* < 0.05). After nCRT, the presence of EMVI was significantly high in tumors with lymphovascular and perineural tumor growth (*p* = 0.034). EMVI status was an independent adverse prognostic factor for disease-free survival [hazard ratio (HR) 7.0, 95% confidence interval (CI) 2.3–21.8; *p* =0.001] and overall survival (HR 6.5, 95% CI 2.2–19.1; *p* = 0.001) in the surgery-alone group for node-positive tumors.

**Conclusions:**

In this study of locally advanced > pT3/N0-3 EC patients, EMVI was present in 23.5% of patients in the surgery-alone group and in 21.6% of patients after nCRT. EMVI was an independent adverse prognostic factor in patients after surgery alone.

Invasion of tumor cells into blood vessels is an important expression of the metastatic potency of malignant tumors. In esophageal cancer (EC), distant metastases are common and seem to be associated with venous invasion (VI).^1^,^2^ Current TNM classifications recognize lymphovascular invasion (LVI) as a prognostic factor. 3,4 Hence, it may be important to report the type of vascular invasion (VI) of tumor cells during routine pathologic workup in EC. Both the Association of Directors of Anatomic and Surgical Pathology (ADASP) and the College of American Pathologists (CAP) stress that extramural venous invasion (EMVI) is an independent predictor of poor prognosis in colorectal cancer (CRC).^5^,^6^ EMVI is defined as the microscopic presence of tumor cells in venous blood vessels beyond the muscularis propria (Fig. 1).^6^–^11^

**Fig. 1:**
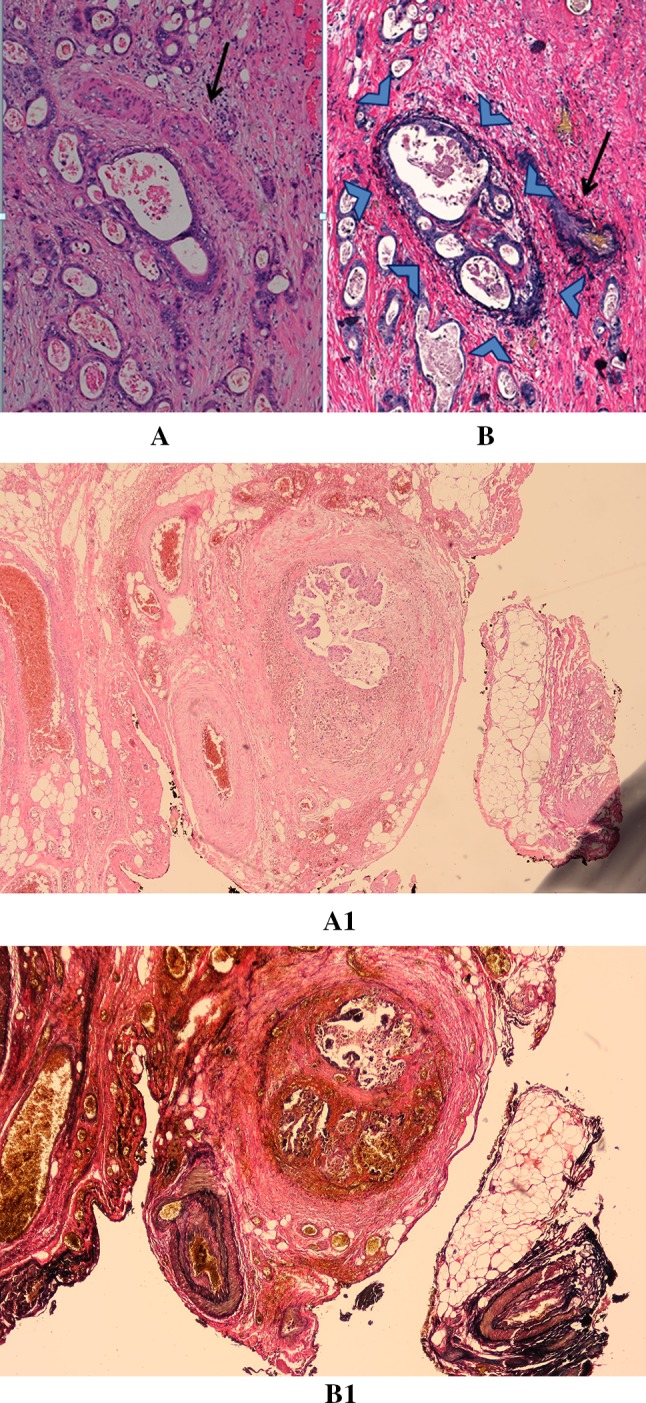
‘Orphan artery’ sign (arrow) suspicious for extramural venous invasion identified on a hematoxylin and eosin slide in esophageal cancer resection (A/A1). The deeper section of the corresponding tumor specimen stained with Elastica van Gieson shows the black-stained elastic fibers around the vein (arrowheads) next to the artery (arrow), confirming the presence of tumor cells in an extramural vein (B/B1)

The prevalence of EMVI in CRC resection specimens is approximately 28%.^6^ In contrast to CRC, the prevalence and prognostic significance of EMVI in EC has not been well studied.^9^ In general, most of the studies in EC were performed to differentiate VI from lymphatic invasion.

Presently, neither the Royal College of Pathologists (RCP) nor the CAP protocol requires differentiation of intramural VI (IMVI), EMVI, and lymphatic invasion in esophageal resection specimens.^9^ To this end, the objectives of our study were to determine the prevalence and assess the prognostic significance of EMVI, confirmed or excluded by Elastica van Gieson (EVG) staining of the resection specimens in patients with pathological T3 or higher EC treated by surgery alone, and those with neoadjuvant chemoradiotherapy (nCRT) followed by surgery.

## Materials and Methods

### Patients

EMVI was retrospectively analyzed on prospectively collected data of 182 consecutive patients with a curative resectable EC who underwent surgery alone between 2000 and 2014. Excluded were 98 patients with a pathological stage ≤ T1–T2 EC and/or cardia-gastroesophageal junction (GEJ; type II/III) tumors, which are frequently considered as gastric cancer. All patients were aged ≥ 18 years and were staged according to a standard protocol with 18F-fluorodeoxyglucose positron emission tomography (FDG-PET), computed tomography (CT), and endoscopic ultrasonography (EUS). To evaluate the impact of EMVI after current treatment with nCRT plus surgery according to the CROSS (ChemoRadiotherapy for Oesophageal cancer followed by Surgery Study),^12^ we also examined 37 of the 54 patients with a pathological T3 EC who met the same inclusion criteria, with the exception of treatment with nCRT between 2012 and 2015. This study was performed according to the rules of the National Health Care, with approval from the Institutional Ethics Board.

### Methods

#### Histopathology Assessment

For each patient, archival slides of the tumor resection specimens were reviewed by two gastrointestinal pathologists. EMVI was scored as the presence of tumor cells within venous structures beyond the muscularis propria, as characterized by the ‘protruding tongue’ and the ‘orphan artery’ signs. The ‘protruding tongue’ sign is defined as a tongue-like protrusion of tumor extending from the deepest invasive front into the surrounding peri-esophageal fat,^9^ and the ‘orphan artery’ sign is observed when tumor invasion is present as a circumscribed tumor nodule near a muscularized artery without invading accompanying veins on hematoxylin and eosin (H&E) slides (Fig. 1).^6^,^13^ In all EMVI suspected cases on H&E slides, additional EVG staining was performed to confirm or exclude the presence of EMVI. The elastic lamina within medium to large vessels can be seen as black elastic fibers in EVG-stained slides, which can distinguish lymphatic vessels from veins. EVG staining was performed according to a standard protocol.^11^ Slides of 4–5 µm were performed from the corresponding tumor in paraffin blocks. One to three tumor slides from cases suspicious for EMVI were additionally stained with EVG.

Other examined pathological parameters were T stage (T3/T4), N stage, tumor length, histologic type, tumor differentiation grade (well/moderate vs. poor/signet cell/mucinous), perineural invasion (PNI), and circumferential resection margin (CRM) involvement. PNI was defined as tumor cell infiltration in any layer of the nerve sheath, while both LVI and VI were defined as the presence of tumor cells within an endothelium-lined space without underlying muscular walls. CRM was assessed according to the RCP definition, which defines microscopic tumor cells within 1 mm of the CRM as involved (R1 resection).

#### Follow-Up

Patients were evaluated postoperatively every 3 months for the first year, every 6 months for the second and third years, and yearly thereafter. If recurrence was clinically suspected, patients underwent CT or PET/CT scans of the chest/abdomen, and endoscopy for confirmation of locoregional recurrence. Recurrent disease was either proven cytologically/histologically or when unequivocally present on radiologic imaging.

#### Statistics

Fisher’s exact test was used to compare characteristics of patients with and without EMVI, and the log-rank test was used for univariable survival analyses of disease-free (DFS) and overall survival (OS). DFS was defined as the time from surgery to the date of recurrence or death. The prognostic value of different factors was first examined in univariable analyses. Clinically relevant factors with a *p* value < 0.2 in univariable analysis were included in the Cox regression multivariable analysis. Survival curves were calculated using the Kaplan–Meier method. Patients were censored at the last point of known contact during follow-up without acquiring the outcomes of interest. The backward conditional Cox regression model was used to delineate significant prognostic factors for survival. Hazard ratios (HR) and 95% confidence intervals (CI) were generated, and a *p* value < 0.05 was considered significant in the multivariable analysis. Statistical analysis was performed using SPSS 20.0 software (IBM Corporation, Armonk, NY, USA).

## Results

### Patient and Tumor Characteristics

#### Surgery-Alone Group

Of the 84 included patients with pathological ≥T3 EC, EMVI was suspected in 47 patients (55.9%) on review of H&E-stained slides. In three suspicious EMVI cases, EVG staining could not be performed due to the loss of the area of interest in additional slides, and these patients were therefore excluded from further analysis. The median age of the remaining 81 patients was 68 years (range 50–85). Tumor-negative CRM of > 1 mm (R0) was seen in 42 (51.9%) patients, and EMVI could be confirmed on EVG-stained slides in 19 (23.5%) cases. The relation of EMVI with the examined tumor characteristics are presented in Table 1. The presence of EMVI was significantly high in tumors located in the mid-esophagus (57.1% vs. 16.4% in the distal esophagus; *p* = 0.003) and in squamous cell carcinomas [SSC; 42.9% vs. 16.7% in adenocarcinoma (AC); *p* = 0.033]. Moreover, the presence of EMVI was significantly higher in patients with LVI-positive tumor (*p* = 0.006). EMVI showed no relationship with pathological (p)T stage and pN stage, tumor length or differentiation grade, or with PNI or CRM involvement (Table 1).

**Table 1:** Cohort demographics by the presence or absence of EMVI in the surgery-alone group

Pathological characteristics	Total [*N* = 81] (100%)	EMVI-positive [*N* = 19] (23.5%)	EMVI-negative [*N* = 62] (76.5%)	*p* value
Localization				
Middle	14 (17.3)	8 (42.1)	6 (9.7)	**0.003**
Distal	67 (82.7)	11 (57.9)	56 (90.3)	
Histologic type				
AC	60 (74.0)	10 (52.6)	50 (80.6)	**0.033**
SCC	21 (26.0)	9 (47.4)	12 (19.4)	
T stage				
T3	74 (91.3)	19 (100)	55 (88.7)	0.190
T4	7 (8.6)	0 (0)	7 (11.3)	
N stage				
Present	59 (72.8)	13 (68.4)	46 (74.2)	0.769
Absent	22 (27.2)	6 (31.6)	16 (25.8)	
Differentiation grade				
Well/moderated	51 (63.0)	15 (78.9)	36 (58.1)	0.113
Poor signet/mucinous	30 (37.0)	4 (21.0)	26 (41.9)	
Tumor length, cm^a^				
> 3	64 (81)	18 (94.7)	46 (76.7)	0.154
≤ 3	15 (19)	1 (5.3)	14 (23.3)	
Perineural invasion				
Present	51 (63.0)	14 (73.7)	37 (59.7)	0.537
Absent	30 (37.0)	5 (26.3)	25 (40.3)	
Lymphovascular invasion				
Present	29 (35.8)	12 (63.2)	17 (27.4)	**0.006**
Absent	52 (64.2)	7 (36.8)	45 (72.6)	
CRM				
Positive, ≤ 1 mm	39 (48.1)	7 (36.8)	32 (51.6)	0.302
Negative, > 1 mm	42 (51.9)	12 (63.2)	30 (48.4)	

Bold values are statistically significant (*p* < 0.05)

*CRM* circumferential resection margin, *EMVI* extramural venous invasion, *AC* adenocarcinoma, *SCC* squamous cell carcinoma

^a^Two missing values

#### Neoadjuvant Treatment Group

Of the 37 patients with pathological T3 EC enrolled in this study, EMVI was suspected in 19 (51.4%) patients on review of H&E-stained slides (Table 2). Tumor-negative CRM of > 1 mm (R0) was seen in 33 patients (89.2%), and EMVI could be confirmed on EVG-stained slides in 8 of the 19 cases (42.1%). In relation to the examined tumor characteristics (Tables 3, 4), EMVI was only significant in tumors with LVI and perineural tumor growth (*p* = 0.034).

**Table 2:** Univariable analyses with regard to DFS and OS in 74 patients with ≥ pT3 in the surgery-alone group

Factor	DFS		OS	
	HR (95% CI)	*p* value	HR (95% CI)	*p* value
Sex (male/female)	0.6 (0.2–1.2)	**0.155**	0.6 (0.3–1.3)	0.227
Age	0.7 (0.3–1.8)	0.548	0.9 (0.4–2.0)	0.805
Tumor length (≤ 3/> 3 cm)	0.9 (0.9–1.0)	0.291	1.0 (0.9–1.0)	0.264
Localization (mid/distal)	0.9 (0.6–1.3)	0.721	0.9 (0.4–1.9)	0.884
Histology (SCC/AC)	0.7 (0.4–1.5)	0.450	0.6 (0.7–1.3)	0.238
pT stage (T3/T4)	2.9 (1.0–8.3)	**0.047**	4.0 (1.6–9.8)	**0.002**
pN1-3	1.7 (1.2–2.4)	**0.002**	1.5 (1.1–2.1)	**0.004**
Differentiation grade (good/moderate vs. poor/signet cell/mucinous)	0.8 (0.4–1.5)	0.641	0.7 (0.4–1.4)	0.367
Perineural growth (present/absent)	1.3 (0.7–2.3)	0.411	1.0 (0.6–1.8)	0.937
Lymphovascular invasion (present/absent)	1.1 (0.6–2.1)	0.649	1.2 (0.7–2.1)	0.491
CRM (positive/negative)	2.4 (1.4–4.3)	**0.002**	2.0 (1.2–3.4)	**0.012**
EMVI (present/absent)	1.4 (0.7–2.7)	0.305	1.5 (0.8–2.7)	0.236
EMVI/N (nodal status)	1.8 (1.3–2.6)	**0.001**	1.8 (1.3–2.6)	**< 0.001**
EMVI −/N −	Ref.		Ref.	
EMVI +/N −	1.8 (0.4–7.7)	0.413	1.9 (0.4–8.0)	0.377
EMVI −/N +	3.6 (1.4–9.3)	**0.008**	3.9 (1.5–9.9)	**0.005**
EMVI +/N +	6.1 (2.0–18.6)	**0.002**	6.3 (2.1–18.3)	**0.001**

Bold indicates *p* value < 0.2 in univariable analysis were included in the Cox regression multivariable analysis

*DFS* disease-free survival, *OS* overall survival, *p* pathological, *CRM* circumferential resection margin, *EMVI* extramural venous invasion, *HR* hazard ratio, *CI* confidence interval, *SCC* squamous cell carcinoma, *AC* adenocarcinoma

**Table 3:** Multivariable analyses (backward conditional Cox regression model) with regard to DFS and OS in 74 patients with ≥ pT3 esophageal cancer in the surgery-alone group

Factor	DFS		OS	
	HR (95% CI)	*p* value	HR (95% CI)	*p* value
Sex	1.0 (10.0–1.0**)**	0.983	**–**	
pT stage (T3 vs. T4)	2.1 (0.7–6.2)	0.183	3.7 (1.5–9.2)	**0.006**
pN1-3	0.9 (0.5–1.7)	0.793	0.7 (0.4–1.3)	0.210
CRM [positive/negative]	2.4 (1.3–4.6)	**0.005**	1.5 (0.9–2.7)	0.156
EMVI/N (nodal status)	1.8 (1.3–2.7)	**0.001**	1.8 (1.3–2.6)	**< 0.001**
EMVI −/N −	Ref.		Ref.	
EMVI +/N −	2.5 (0.6–11.1)	0.214	1.9 (0.5–8.2)	0.364
EMVI −/N +	3.0 (1.1–7.8)	**0.024**	3.5 (1.3–9.1)	**0.010**
EMVI +/N +	7.0 (2.3–21.8)	**0.001**	6.5 (2.2–19.1)	**0.001**

Bold values are statistically significant (*p* < 0.05)

*DFS* disease-free survival, *OS* overall survival, *p* pathological, *CRM* circumferential resection margin, *EMVI* extramural venous invasion, *HR* hazard ratio, *CI* confidence interval

**Table 4:** Cohort demographics by the presence or absence of EMVI in the neoadjuvant chemoradiotherapy group

Pathological characteristics	Total [*N* = 37] (100%)	EMVI-positive [*N* =8] (21.6%)	EMVI-negative [*N* = 29] (78.4%)	*p* value
Localization				
Middle	1 (2.7)	0 (0)	1 (3.4)	0.784
Distal	36 (97.3)	8 (100)	28 (96.6)	
Histologic type				
AC	35 (94.6)	7 (87.5)	28 (96.6)	0.390
SCC	2 (5.4)	1 (12.5)	1 (3.4)	
T stage				
T3	37 (100)	8 (100)	29 (100)	–
N stage				
Present	17 (45.9)	4 (50)	13 (44.8)	0.553
Absent	20 (54.1)	4 (50)	16 (55.2)	
Differentiation grade				
Well/moderated	23 (62.2)	5 (62.5)	18 (62.1)	0.657
Poor signet/mucinous	14 (37.8)	3 (37.5)	11 (37.9)	
Tumor length, cm				
> 3	31 (83.8)	7 (87.5)	24 (82.8)	0.613
≤ 3	6 (16.2)	1 (3.5)	5 (17.2)	
Perineural invasion				
Present	15 (40.5)	6 (75.0)	9 (31.0)	**0.034**
Absent	22 (59.5)	2 (25.0)	20 (68.9)	
Lymphovascular invasion				
Present	15 (40.5)	6 (75.0)	9 (31.0)	**0.034**
Absent	22 (59.5)	2 (25.0)	20 (68.9)	
CRM				
Positive ≤ 1 mm	4 (10.8)	1 (12.5)	3 (10.3)	0.640
Negative > 1 mm	33 (89.2)	7 (87.5)	26 (89.6)	
Mandard classification				
TRG2/3	24 (64.9)	4 (50)	20 (68.9)	0.278
TRG4/5	13 (35.1)	4 (50)	9 (31.0)	

Bold values are statistically significant (*p* < 0.05)

*CRM* circumferential resection margin, *EMVI* extramural venous invasion, *AC* adenocarcinoma, *SCC* squamous cell carcinoma, *TRG* tumor regression grade

### Survival Analysis

#### Surgery-Alone Group

Follow-up data were available in 74 of the 81 examined patients. Excluded from survival analysis were six patients who died < 90 days after surgery and one patient with a simultaneous colon carcinoma. In the remaining 74 resected esophageal tumors, EMVI was confirmed in 17 of 37 (46%) suspected cases. Median DFS (22 vs. 22 months; *p* = 0.297) and OS (23 vs. 26 months; *p* = 0.226) were not different among patients with or without EMVI. Distant recurrence-free survival was also not different in both groups (22 vs. 25 months; *p* = 0.306).

**Fig. 2:**
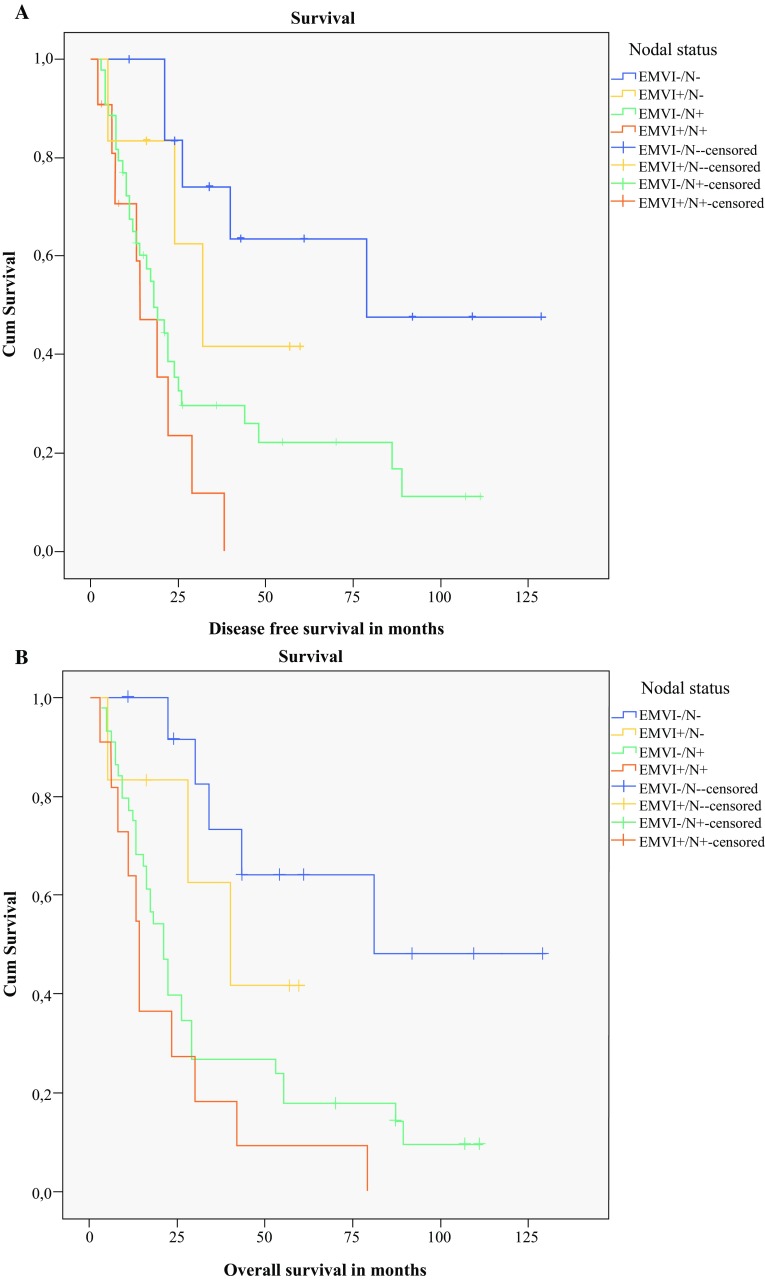
**a** Kaplan–Meier survival curve for disease-free survival stratified by EMVI and lymph node status after surgery alone (*p* = 0.004). **b** Kaplan–Meier survival curve for overall survival stratified by EMVI and lymph node status after surgery alone (*p* = 0.002). *EMVI* extramural venous invasion, *Cum* cumulative

In the univariable analysis (Table 2), independent prognostic factors associated with DFS were gender, pT3 stage, pN1-3, positive CRM and EMVI/N (nodal status). The backward conditional Cox regression multivariable analysis (Table 3) showed that positive CRM (HR 2.4, 95% CI 1.3–4.6) and EMVI/N (nodal status) [HR 1.8, 95% CI 1.3–2.7] were independent prognostic factors for DFS. The median DFS for EMVI and nodal status were significantly different: EMVI −/N − : 79 months; EMVI +/N − : 32 months; EMVI −/N + : 18 months; EMVI +/N + : 14 months (Fig. 2a).

In the univariable analysis (Table 2), independent prognostic factors associated with OS were pT, pN stage, CRM and EMVI/N (nodal status). The backward conditional multivariable Cox regression analysis showed that independent prognostic factors for OS were pT stage (HR 3.7, 95% CI 1.5–9.2) and EMVI/N (nodal status) [HR 1.8, 95% CI 1.3–2.6] (Table 3). The median OS for EMVI and nodal status were significantly different: EMVI −/N − : 81 months; EMVI +/N − : 40 (14–65) months; EMVI −/N + : 21 (16–25) months; EMVI +/N + : 14 (10–17) months (Fig. 2b).

When adjusted for histologic type, EMVI was significantly prognostic for DFS in SCCs (HR 5.0, 95% CI 1.0–23.8; *p* = 0.043) and for OS in ACs after surgery alone (HR 2.5, 95% CI 1.1–5.6; *p* = 0.031) [data not shown].

#### Neoadjuvant Treatment Group

Three of the 37 included patients with pathological T3 EC died < 90 days after surgery and were excluded from survival analysis. In the remaining 34 esophageal tumors, EMVI was confirmed in 8 of 26 (30.7%) suspected cases. Both DFS (15 vs. 26 months; *p* = 0.827) and OS (median 17 vs. 34 months; *p* = 0.954) were not different among patients with or without EMVI. Both the univariable analyses and backward conditional multivariable Cox regression analysis have shown no independent prognostic factors associated with DFS (Tables 5, 6). The median DFS for EMVI and nodal status were not significantly different: EMVI −/N − : 41 months; EMVI +/N − : 13 months; EMVI −/N + : 16 months; EMVI +/N + : 15 months after nCRT.

**Table 5:** Univariable analyses with regard to DFS and OS in 34 patients with pT3 esophageal cancer after neoadjuvant chemoradiotherapy

Factor	DFS		OS	
	HR (95% CI)	*p* value	HR (95% CI)	*p* value
Sex (male/female)	0.7 (0.2–3.1)	0.653	0.8 (0.2–3.4)	0.732
Age	0.2 (0.0–1.5)	**0.120**	0.4 (0.1–1.9)	0.267
Tumor length (≤ 3/> 3 cm)	1.1 (0.3–4.0)	0.942	1.4 (0.4–4.9)	0.568
Localization (mid/distal)	0.5 (0.1–4.1)	0.557	0.4 (0.0–2.8)	0.327
Histology (SCC/AC)	0.5 (0.1–4.0)	0.544	0.6 (0.0–4.2)	0.327
pN1-3	1.9 (0.8–4.6)	**0.157**	2.0 (0.8–5.0)	**0.129**
Differentiation grade (good/moderate vs. poor/signet cell/mucinous)	1.0 (0.4–2.3)	0.942	1.0 (0.4–2.5)	0.966
Perineural growth (present/absent)	1.1 (0.5–2.6)	0.840	1.2 (0.5–3.0)	0.656
Lymphovascular invasion (present/absent)	1.1 (0.4–2.5)	0.885	0.9 (0.4–2.3)	0.879
CRM (positive/negative)	2.3 (0.7–7.3)	**0.155**	3.7 (1.1–12.4)	**0.034**
EMVI (present/absent)	1.1 (0.4–3.1)	0.829	0.9 (0.3–2.9)	0.955
EMVI/N (nodal status)	1.3 (0.9–19)	**0.173**	1.3 (0.9–2.0)	**0.174**
EMVI −/N −	Ref.		Ref.	
EMVI +/N −	0.9 (0.2–4.2)	0.997	1.0 (0.2–4.9)	0.961
EMVI −/N +	1.8 (0.7–4.9)	0.236	2.1 (0.8–5.7)	**0.148**
EMVI +/N +	2.1 (0.5–8.3)	0.287	1.8 (0.4–9.1)	0.463
Mandard classification TRG2/3/TRG4/5	1.4 (0.6–3.4)	0.401	1.2 (0.5–3.0)	0.667

Bold indicates *p* value < 0.2 in univariable analysis were included in the Cox regression multivariable analysis

*DFS* disease-free survival, *OS* overall survival, *p* pathological, *CRM* circumferential resection margin, *EMVI* extramural venous invasion, *HR* hazard ratio, *CI* confidence interval, *AC* adenocarcinoma, *SCC* squamous cell carcinoma, *TRG* tumor regression grade

**Table 6:** Multivariable analyses (backward conditional Cox regression model) with regard to DFS and OS in 34 patients with pT3 esophageal cancer after neoadjuvant chemoradiotherapy

Factor	DFS		OS	
	HR (95% CI)	*p* value	HR (95% CI)	*p* value
Age	0.2 (0.0–1.5)	0.120	–	
pN1-3	1.3 (0.9–2.0)	0.174	1.6 (1.1–2.5)	**0.020**
CRM (positive/negative)	1.4 (0.4–5.3)	0.572	2.1 (0.6–7.9)	0.272
EMVI/N (nodal status)	0.7 (0.3–1.9)	0.524	0.7 (0.3–1.6)	0.460
EMVI −/N −	Ref.		Ref.	
EMVI +/N −	1.1 (0.2–5.4)	0.888	1.0 (0.2–4.9)	0.967
EMVI −/N +	0.5 (0.1–3.9)	0.512	0.3 (0.0–3.5)	0.366
EMVI +/N +	0.6 (0.1–5.4)	0.692	0.3 (0.0–3.9)	0.382

Bold value is statistically significant (*p* < 0.05)

*DFS* disease-free survival, *OS* overall survival, *p* pathological, *CRM* circumferential resection margin, *EMVI* extramural venous invasion, *HR* hazard ratio, *CI* confidence interval

In the univariable analyses, a positive CRM was the only independent prognostic factor associated with OS (HR 3.7, 95% CI 1.1–12.4) (Table 5). The backward conditional Cox regression multivariable analysis has shown that nodal involvement (HR 1.6, 95% CI 1.1–2.5) was the only independent prognostic for OS (Table 6). The median OS for EMVI and nodal status were not different: EMVI −/N − : 37 months; EMVI +/N − : 15 months; EMVI −/N + : 18 months; EMVI +/N + : 17 months after nCRT.

## Discussion

In EC, the prevalence and significance of EMVI has not been well studied. In this study, EMVI was present in approximately one-quarter of patients with a pathologically staged T3 or higher EC after surgery alone, and in 21.6% of patients after nCRT. The rate of EMVI in the surgery-alone group was higher in mid-esophageal carcinomas (42.1% vs. 9.7%) and SCCs (47.4% vs. 19.4%), as well as in tumors with LVI (63.2% vs. 27.4%). In the nCRT group, the numbers regarding type and tumor side were too small to draw clear conclusions. However, EMVI was significantly higher in tumors with perineural tumor growth and those with LVI—both 75% vs. 31%, respectively.

More or less the same percentages are found as in CRC, where EMVI is reported in approximately 28% (range 13.4–31.4%) of the resection specimens.^6^ As in CRC, EMVI was common in high T- and N-staged EC.^6^ However, there is wide variability in the assessment of VI. Several reports regarding current practice and agreements among pathologists showed a higher detection rate in university hospitals with experienced gastrointestinal pathologists and the benefits of routine use of additional elastin stain. Moreover, accurate detection and quality in reporting of VI had shown a prognostic significance on cancer survival.^14^,^15^

Most reports in EC have only described the presence of VI in general, without further distinction of IMVI and EMVI.^9^,^16^–^18^ Although EMVI is routinely diagnosed by the presence of key hallmarks, i.e. the ‘orphan artery’ and ‘protruding tongue’ signs, accurate detection of EMVI can be problematic. Therefore, separate reporting of venous and small vessel invasion (VI) and notification of EMVI has been recommended in pathological guidelines for CRC.^5^,^19^ EMVI observation can be hampered when the muscular wall of the veins is obliterated beyond morphologic recognition in specimens after surgery alone or when altered by the use of nCRT due to increased vessel fibrosis and destructed vessel wall architecture. This may result in under-identification. Therefore, elastin stain, known as the EVG of vessel walls, is generally advocated to solve the majority of these difficult cases.^13^ EVG stain judged as absent or present has shown to double the EMVI detection rates, with increased interobserver agreement between pathologists.^19^–^21^

For the identification of VI in esophageal AC, Castonguay et al. used Movat stains, which is more or less equal to EVG staining.^9^ Both are useful for visualizing connective tissue and elastic fibers. Due to the damage and loss of endothelium, immunohistochemistry, including CD31 and CD34, is often inconclusive. However, in some instances, the only residual clue of VI is a layer of elastin around a round nest of neoplastic cells, which can only be confirmed reliably with elastin stain.^21^–^24^

As in CRC, we found that EMVI in EC should be considered as a potential high-risk factor in developing metastatic disease. Consequently, as in CRC, we also observed a significantly shorter DFS in the surgery-alone group (HR 2.4, 95% CI 1.1–4.9) in EMVI-positive EC compared with EMVI-negative tumors (median 25 and 48 months); however, in the nCRT group, the presence of EMVI was not independently associated with survival. The clinical utility in this nCRT group was probably lacking due to a potential bias from case mix with low squamous cell cancers, therefore EMVI should be investigated in a larger study group. Another explanation may be the power of our study group, which was too small or was based on the selection of non-responders to nCRT. Failure of EMVI to regress after nCRT may indicate a lack of response, as shown by the association of EMVI changes on magnetic resonance imaging (MRI) with survival outcomes in CRC, and a potential positive effect of nCRT on microscopic distant disease.^13^,^25^–^27^ Regression of EMVI following neoadjuvant treatment that results in vessel fibrosis can be used as a predictive imaging biomarker in several gastrointestinal cancers.^25^,^26^ Moreover, in rectal cancer, the presence of MRI-related EMVI (mrEMVI) within or beyond the mesorectal fat was used to identify high-risk patients who may benefit from neoadjuvant treatment, whereas among patients with stage III gastric cancer, contrast-enhanced multiple-row detector CT might also depict EMVI.^25^–^27^ Whether the presence and grading of EMVI following nCRT can be accurately assessed on radiologic imaging should be investigated in ongoing or future diffusion-weighted imaging/MRI studies.

To our knowledge, this is the first study comparing outcomes of EMVI among pathological T3 or higher stages in a relatively large group of EC resection specimens in a single, high-volume institute. The usual limitations of retrospective studies and the relatively small sample size of EMVI hinder our ability to draw definite conclusions on the significance of EMVI, especially after nCRT. However, there was no difference in histology type for DFS after surgery alone. The prognostic impact of EMVI after nCRT in SCC is still unclear. EMVI is a histopathologic feature associated with an increased risk of recurrences, and, based on our study, it should be considered as a routine part of pathological reports of resection specimens of EC patients. However, its value should be studied prospectively in larger series and following nCRT in different tumor stages and different histological types.

## Conclusion

In this study, EMVI was present in 23.5% of patients in the surgery-alone group in 21.6% of patients after nCRT. EMVI was an independently adverse prognostic factor in patients after surgery alone. Therefore, EMVI should be considered as part of the routine histopathology workup, with an accessible use of elastin stains.
